# Surgery

**Published:** 1903-12

**Authors:** James Swain


### SURGERY.

**The operative surgery of the pancreas** is so recent that a summary of what has been done may well occupy our attention, and papers by Dr. Deaver[2] and Professor Von Mikulicz-Radecki[3] may be taken as a basis for a statement of the present position of the subject. We need not concern ourselves with the treatment of pancreatic cysts, which is now well known; but in regard to trauma, inflammatory conditions and new growths, it is only within the past few years that surgical treatment has been undertaken.

1 "A Report of Seven Operations for Brain Tumours and Cysts." *J. Am. M. Ass.*, 1901, xxxvi. 302.

2 *Am. J. M. Sc.*, 1903, cxxv. 187. 3 *Ann. Surg.*, 1903, xxxviii. 1.

There are many reasons for the late development of pancreatic surgery. The pancreas is so well protected by its position that it is rarely injured, and even when such is the case there usually exist severe complicating injuries of neighbouring organs, so that the patient dies before the surgeon has an opportunity of interfering, or the lesion of the pancreas is easily overlooked.

Then there is the difficulty of diagnosis and the indefinite character of the subjective symptoms; for signs of functional disturbance of this important organ do not appear until the greater part of the gland is affected, and the surgeon has no longer any right to interfere when pancreatic diabetes has developed. It therefore follows that most cases are operated upon when the diagnosis is only probable, and only after the abdomen is opened can a differential diagnosis be made in many instances.

Moreover, in addition to the great danger from hemorrhage, there is a still greater one that is due to the special secretion of the gland leaking from the injured parenchyma in larger or smaller quantities and diminishing the resisting power of the peritoneum. Statistics show that in cases of injury of the pancreas tampon drainage has achieved better results than the closure of the abdomen without drainage, and it may therefore be concluded that wherever the pancreatic tissue has been exposed at all, the abdominal cavity must be tamponed and drainage established.

**Pancreatitis** is best divided clinically into the acute, subacute and chronic varieties. The classification of Fitz into hemorrhagic, suppurative and gangrenous pancreatitis rather represents different stages of the disease, with a common ætiology. Bacterial invasion undoubtedly plays an important part in acute pancreatitis, but it does not entirely explain the singularly severe symptoms. A severe hemorrhage from the pancreatic and retroperitoneal tissue occurs in these cases as a result of a peculiar dyscrasia of the patient, accompanied by hemorrhagic peritoneal exudation, which is not of bacterial origin. There can be no doubt that there is some special cause for this phenomenon, and it may best be attributed to the action of the pancreatic and fat-splitting ferments upon the gland which is the seat of a hemorrhage or has been otherwise injured. Alcoholism, arterio-sclerosis, syphilis and fatty degeneration must be regarded as predisposing factors of pancreatitis. Gastro-duodenal catarrh, with extension to the duct and followed by bacterial invasion, may be a factor; and ulcer and cancer of the stomach, pylorus or duodenum, and zymotic diseases, such as enteric fever, mumps and influenza, are the determining causes as stated by Robson and Moynihan.[1]

Biliary and pancreatic calculi are certain causes of pan-

[1] Robson and Moynihan, *Diseases of the Pancreas*, 1903.

creatitis. This has been observed clinically and produced experimentally. **Acute pancreatitis** is characterized by the sudden onset of epigastric pain, accompanied by prostration. Vomiting is an early and severe symptom; there is a tender swelling in the epigastrium, tympanitic on percussion, and constipation is so marked that a diagnosis of acute intestinal obstruction has frequently been made. Collapse ending in delirium and death may rapidly follow. Intestinal obstruction is the usual diagnosis made, as in a case referred to by the writer in this *Journal* about two years ago.[1] The obstruction, however, is not so absolute, and repeated enemata before peritonitis has supervened finally restore the peristalsis of the bowel. In favour of pancreatic disease is the history of previous attacks.

The treatment of these acute cases is still open to discussion. In true pancreatic apoplexy, owing to existing serious constitutional dyscrasia, a surgical procedure would hardly be justifiable, and on the other hand some of the milder cases recover without operation. But we know that hemorrhage into the pancreas may cause foci of gangrene and suppuration, and for purposes of treatment it is better to regard the majority of cases of acute pancreatitis as an acute phlegmon which, on account of the peculiar nature of the tissue, runs an unusually severe course; and as in an ordinary phlegmon, so in the pancreas, the only rational therapy—except in the very mild or very severe cases to which reference has been made—is to open the focus of infection and to empty and drain the toxic and infectious exudate. Gauze tampons will best combat the tendency to fatal hemorrhage. It is in the very early stage—before the formation of abscess—that surgeons are not agreed as to the desirability of operation; but inasmuch as there are many recorded cases where benefit has been derived from the removal of the hemorrhagic exudate, Mikulicz-Radecki[2] considers the following points to be in favour of surgical interference:—(1) The operation, under local anæsthesia, can be performed so easily that we can employ it even in collapsed patients without running any great additional risks. (2) Emptying of the peritoneal exudate and thorough flushing of the abdominal cavity with normal saline solution are surely of great benefit to the patient, more especially when the peritoneal cavity is drained later on. (3) An artificial anus should be established only when intestinal paralysis exists. By active interference in the acute stage we not only desire to overcome the septic condition to which most of the patients succumb, but also to ward off necrosis and sequestration of large portions of the gland occurring in many cases. The treatment of those cases which have run on to suppuration and gangrene consists in laparotomy, evacuation of pus, and drainage of the purulent foci.

In the **subacute forms of pancreatitis** the onset is more

[1] *Bristol M.-Chir. J.*, 1902 xx. 40. [2] *Loc. cit.*

gradual, the pain is less severe and the vomiting is not so violent. Here the surgeon has time to observe the case more carefully. The treatment at first may consist in the administration of morphine to quiet the pain, the use of stimulants to combat the depression, and the use of lavage and rectal feeding to relieve the vomiting. If suppuration occurs, evacuation and drainage of the abscess must be resorted to.

**Chronic indurative pancreatitis** is probably a more common disease than is usually supposed. Many cases diagnosed as malignant disease of the head of the pancreas are of this character. Opie's[1] conclusions so well express our knowledge of this condition that they may here be quoted:—(1) Chronic interstitial pancreatitis is slightly more frequent in males than in females. Two-thirds of the total number of cases occur between the ages of forty and sixty years. (2) The most frequent cause of chronic pancreatitis is obstruction of the duct of Wirsung, due to pancreatic calculi, to biliary calculi in the terminal part of the common bile-duct, or to carcinoma invading the head or body of the gland. Duct obstruction may be followed by the invasion of bacteria, which take part in the production of the resulting lesion. (3) Ascending infection of the unobstructed duct of Wirsung may follow an acute lesion of the duodenum or of the bile passages, and may cause chronic inflammation. In cases which have given a history of long, persistent vomiting, chronic diffuse pancreatitis may be found on autopsy, and is probably the result of an ascending infection of the gland. (4) General or local tuberculosis is occasionally accompanied by chronic diffuse pancreatitis, affecting chiefly the interstitial tissue of the gland. (5) Chronic interstitial pancreatitis is not infrequently dependent upon the same ætiological factors, notably alcohol, which produce cirrhosis of the liver, and in about one-fourth of the cases the two lesions are associated. (6) Following duct obstruction and ascending infection, the lesion affects principally the interlobular tissue, only secondarily invading the lobular tissue and sparing the islands of Langerhans. Diabetes results only when the lesion is far advanced. (7) Accompanying the so-called atrophic or Laennec's cirrhosis of the liver, the pancreas is sometimes the seat of diffuse chronic inflammation, characterized by diffuse proliferation of the interacinar tissue, which invades the islands of Langerhans. (8) Interacinar pancreatitis is usually accompanied by diabetes mellitus. When diabetes is absent the lesion is of such slight intensity that the islands of Langerhans are little implicated. (The islands of Langerhans are groups of polygonal cells in the parenchyma of the lobules. —J. S.) The treatment of chronic pancreatitis consists in the relief of tension by opening and draining the gall-bladder with the formation of a biliary fistula. Of thirty-six cases reported, thirteen recovered and five proved fatal. If, however, a definite

[1] *Am. J. M. Sc.*, 1902, cxxiii. 868.

cause can be found—such as impacted concretion in the common bile-duct or Wirsung's duct—an attempt must be made to remove it. Fortunately calculi of the pancreas as a cause of this condition are extremely rare.

Of the **new growths occurring in the pancreas,** carcinoma is the most frequent. Sarcoma, adenoma and lymphoma are rare. The head of the organ is most commonly involved in carcinoma, and the disease may be primary or, what is more frequent, secondary to malignant disease of the surrounding structures, especially the stomach. The differential diagnosis of this disease is exceedingly difficult in the early stage and rather easy in the late stage. The development of obstructive jaundice following dyspeptic attacks, with a distended gall-bladder and ever-increasing cachexia, are the most important signs. Death usually occurs within twelve months. Two diseases—viz., common duct obstruction with a gall-stone and chronic interstitial pancreatitis—must be diagnosed, because they are usually cured by a surgical operation, while malignant disease is nearly hopeless (of fourteen cases where a portion of the affected gland was removed there were ten deaths). In the gall-stone affection there is nearly always a history of previous colics, because the stone must have passed the cystic and traversed the common duct before impaction near the head of the pancreas. The gall-bladder is not distended and the jaundice varies in intensity. In chronic pancreatitis the history of gall-stone attacks and its association with dyspepsia must be elicited. The age of the simple affection is earlier, and there is more tenderness. The cachexia is very slight compared to that of carcinoma, and the jaundice is not so intense. The treatment of malignant disease offers little prospect of cure. The risk of injury to blood-vessels, together with shock, gangrene of the duodenum and peritonitis, is very great. The biliary stasis may be relieved by cholecystostomy or cholecystenterostomy.

**X-ray photographs in pathological conditions of the pancreas** would seem to be a development of the near future. According to Mr. Wilbert[1] we can usually find in cases of congested or hypertrophied pancreas a distinct and comparatively dense shadow extending a little to the right of the median line and about midway between the lower end of the sternum and umbilicus. One distinctive feature of the shadow cast by the pancreas is the fact that it is not readily movable with the motion of the diaphragm. By this simple characteristic we are usually able to differentiate between an enlarged or dense head of the pancreas and any pathological change involving the liver or stomach, though it must be borne in mind that adhesions

[1] *Am. J. M. Sc.*, 1903, cxxv. 207.

involving these organs will inhibit, or at least restrict, the natural range of motion.

Another distinctive feature may be established by taking advantage of the fact that the pancreas is located near the posterior wall of the abdomen, and on this account the resulting shadow is readily displaced by changing the relative position of the body, tube and fluorescent screen. For instance, if we move the cause of the shadow above the anode of the tube the shadow is high up, but when moved in the opposite direction it appears to be comparatively lower down.

JAMES SWAIN.

